# Hepatic metabolomics and transcriptomics to study susceptibility to ketosis in response to prepartal nutritional management

**DOI:** 10.1186/s40104-019-0404-z

**Published:** 2019-12-18

**Authors:** Khuram Shahzad, Vincenzo Lopreiato, Yusheng Liang, Erminio Trevisi, Johan S. Osorio, Chuang Xu, Juan J. Loor

**Affiliations:** 1 COMSATS Institute of Information Technology, ChakShahzad, Islamabad, 44000 Pakistan; 2 Department of Animal Sciences and Division of Nutritional Sciences, University of Illinois, Urbana, IL 61801 USA; 3 Istituto di Zootecnica, Facoltà di Scienze Agrarie, Alimentari e Ambientali, Università Cattolica del Sacro Cuore, 29122 Piacenza, Italy; 4 Department of Dairy Science, South Dakota State University, Brookings, SD 57006 USA; 5 College of Animal Science and Veterinary Medicine, Heilongjiang Bayi Agricultural University, Xinyang Rd. 5, Daqing, 163319 China

**Keywords:** Bioinformatics, Ketosis, Metabolomics, Transition cow

## Abstract

**Background:**

Ketosis in dairy cows is associated with body fat mobilization during the peripartal period. Sub-clinical and clinical ketosis arise more frequently in cows that are overfed energy during the entire dry (last 50 to 45 days prior to parturition) or close-up period (last ~ 28 days prepartum).

**Methods:**

A retrospective analysis was performed on 12 cows from a larger cohort that were fed a higher-energy diet [1.54 Mcal/kg of dry matter (DM); 35.9% of DM corn silage and 13% of DM ground corn] during the close-up dry period, of which 6 did not develop clinical ketosis (OVE, 0.83 mmol/L plasma hydroxybutyrate; BHB) and 6 were diagnosed with clinical ketosis (KET, 1.4 mmol/L BHB) during the first week postpartum. A whole-transcriptome bovine microarray (Agilent Technologies) and metabolomics (GC-MS, LC-MS; Metabolon® Inc.) were used to perform transcript and metabolite profiling of liver tissue harvested at − 10 days relative to parturition which allowed to establish potential associations between prepartal transcriptome/metabolome profiles and susceptibility to clinical ketosis postpartum.

**Results:**

Cows in KET had greater (*P* = 0.01) overall body weight between − 2 and 1 week around parturition, but similar body condition score than OVE. Although dry matter intake (DMI) did not differ prepartum, KET cows had lower (*P* < 0.01) DMI and similar milk yield as OVE cows during the first week postpartum. Transcriptome analysis revealed a total of 3065 differentially expressed genes (DEG; *P* ≤ 0.05) in KET. Metabolomics identified 15 out of 313 total biochemical compounds significantly affected (*P* ≤ 0.10) in KET. Among those, greater concentrations (*P* ≤ 0.06, + 2.3-fold) of glycochenodeoxycholate in KET cows also have been detected in humans developing non-alcoholic fatty liver disease. Bioinformatics analysis using the Kyoto Encyclopedia of Genes and Genomes (KEGG) pathway database and the DEG revealed that, among the top 20 most-impacted metabolic pathway categories in KET, 65% were overall downregulated. Those included ‘Metabolism of cofactors and vitamins’, ‘Biosynthesis of other secondary metabolites’, ‘Lipid’, ‘Carbohydrate’, and ‘Glycan biosynthesis and metabolism’. The lower relative concentration of glucose-6-phosphate and marked downregulation of fructose-1,6-bisphosphatase 2 and pyruvate dehydrogenase kinase 4 support a strong impairment in gluconeogenesis in prepartal liver of cows developing KET postpartum. Among the top 20 most-impacted non-metabolic pathways, 85% were downregulated. Pathways such as ‘mTOR signalling’ and ‘Insulin signalling’ were among those. ‘Ribosome’, ‘Nucleotide excision repair’, and ‘Adherens junctions’ were the only upregulated pathways in cows with KET.

**Conclusions:**

The combined data analyses revealed more extensive alterations of the prepartal liver transcriptome than metabolome in cows overfed energy and developing ketosis postpartum. The causative link between these tissue-level adaptations and onset of clinical ketosis needs to be studied further.

## Background

Dairy cows are highly susceptible to developing metabolic disorders such as ketosis during early lactation [1]. Ketosis can affect the profitability of dairy farms in terms of decreased milk production and treatment costs [2]. This disease is associated with negative energy balance (NEB), hepatic lipid accumulation, and increased concentration of blood hydroxybutyrate while blood glucose concentration is decreased [3]. Partial anorexia, lethargic behavior, and body fat mobilization also characterized the ketotic state [4]. The prepartal plane of energy nutrition is highly-correlated with body fat mobilization around parturition, and a greater susceptibility of cows for developing sub-clinical and clinical ketosis [5].

Although a number of studies have used ‘omics’ technology to study alterations in the transcriptome, proteome, and metabolome [6], the majority have addressed changes using liver tissue or plasma/serum in animals diagnosed with the disease [7, 8]. To our knowledge, there are no studies using those tools that have address simultaneously a link between liver tissue omics data and the onset of clinical disease. Thus, the specific objective of the study was to use metabolomics and transcriptomics in liver tissue collected prepartum to explore not only profiles that are associated with postpartum onset of clinical ketosis, but the potential mechanisms at the liver-level that may render cows more susceptible. We used liver tissue harvested prior to parturition from a subset of cows in a previous study from our group [9] that were fed a higher-energy diet during the last 3 weeks prepartum to assess differences in prepartum liver tissue and their association with clinical ketosis outcome in the first week postpartum. Bioinformatics analyses were applied to attempt to integrate transcriptome and metabolome data.

## Methods

All the procedures for this study were approved by The Institutional Animal Care and Use Committee (IACUC) of the University of Illinois at Urbana-Champaign under the protocol No. 09214.

### Experimental design and dietary treatments

The experiment was conducted as a randomized complete block design as explained elsewhere [9] using multiparous Holstein dairy cows. For the present analysis, we used a subset of 12 cows from the full cohort that were overfed and did not develop clinical ketosis (OVE, *n* = 6) or developed clinical ketosis postpartum (KET, *n* = 6). Ketosis was determined during the first 7 days postpartum by testing urine with Ketostix reagent strips (Bayer Corp., Pittsburgh, PA), which measures acetoacetic acid, a ketone. Urine acetoacetic acid > 80 mg/dL was used for the initial diagnosis of KET. All KET cows in the present study had “large ketones” in the urine, went off-feed, and had to be treated by the attending veterinarian with i.v. glucose before returning to the herd. All cows received the same far-off diet [1.24 Mcal/kg of dry matter (DM); 14.3% crude protein (CP)] from − 50 to − 20 days before expected calving, a higher-energy close-up diet (1.54 Mcal/kg of DM; 15.0% CP) from − 21 days to calving, and a common fresh cow lactation diet (1.75 Mcal/kg of DM, 17.5% CP) from calving through 30 days in milk. To increase energy density in the close-up diet, corn silage inclusion went from 33% to 35.9% of DM and ground corn from 4% to 13% of DM, while wheat straw was reduced from 36% to 15.4% of DM. Composition of the total mixed rations, details of blood sampling, and responses in terms of health outcomes and production in the larger cohort of cows have been reported previously [9]. Body weight (BW), body condition score (BCS), dry matter intake (DMI), milk yield, and plasma biomarkers for cows used in the present study are reported herein.

### Liver biopsies and RNA extraction

Liver tissue samples were collected via puncture biopsy [10] from cows under local anesthesia at approximately 07:30 h once prepartum on day − 10 (±3 days) for this study. Details on tissue storage were reported previously [9]. Briefly, liver tissue samples were immediately placed in screw-capped microcentrifuge tubes, snap-frozen in liquid nitrogen, and preserved at − 80 °C until further analysis. Specific details of RNA isolation from liver, primer design, evaluation, and quantitative real time PCR are reported elsewhere [11]. RNA concentration was measured using a Nano-Drop ND-1000 spectrophotometer (Nano-Drop Technologies, Wilmington, DE, USA). The purity of RNA (A260/A280) for all samples was above 2.0. The quality of RNA was evaluated using the Agilent Bioanalyzer system (Agilent 2100 Bioanalyzer, Agilent Technologies, Santa Clara, CA, USA). The average RNA integrity number values for all samples was 7.9.

### Transcriptome and metabolome experiment

The microarray experiment was performed using the ~ 44 K bovine (v2) gene expression Agilent platform. Protocols were exactly as described in our previous publications [12, 13]. Metabolomics analysis was performed using GC-MS/LC-MS by Metabolon® Inc. (Morrisville, NC, USA). Frozen tissue was shipped in dry ice and processed according to company protocols (http://www.metabolon.com).

### Plasma profiling of physiologic biomarkers

Plasma biomarker concentrations were measured at − 12, − 3 and + 3 days relative to parturition using exactly the same commercial kits and protocols as reported in our previous publications [14–16]. Routine evaluation of ketosis was performed by the attending veterinarian during the first 7 days postpartum using urine samples and a reagent strip [17]. Cows that were identified as clinically ketotic (ketones > 80 mg/dL) were immediately treated by the attending veterinarian with intravenous dextrose. Per Animal Care and Use Committee guidelines, once cows were diagnosed they were removed from the experiment and further blood or tissue samples could not be harvested.

### Statistical analysis

The statistical analysis of transcriptome data was performed using 12 microarrays. The data were log-transformed and then corrected for dye and array effects using loess normalization. After data normalization, the mixed procedure of SAS (SAS Institute Inc., Cary, NC) was used to determine differential gene expression. The statistical model included health status as the fixed effect. Differentially expressed genes (DEG) were detected at a False discovery rate *P* ≤ 0.05 and a fold change (FC) of KET versus OVE of ≥1.5.

The statistical analysis of metabolomics data was performed across a total of 313 biochemical compounds identified. The raw values resulting from GC-MS/LC-MS analysis were normalized in terms of raw area counts. The raw values from each biochemical compound were rescaled to set the median value equal to 1. The missing values were imputed with the minimal value. Following log-transformation and imputation of missing values, if any, with the minimal observed value for each compound, the MIXED procedure of SAS was used to identify compounds that differed significantly between OVE and KET. Data for DMI and milk yield, BW and BCS, plasma systemic biomarkers of energy balance, liver function, and immune status were subjected to ANOVA and analyzed using the repeated measures statement in the MIXED procedure of SAS. The model included days or weeks relative to parturition, health status (KET vs. OVE), and their interaction as fixed effects, and cow within health status group as random effect.

### Network analysis and data integration

The network analysis of transcriptomics data was conducted using Ingenuity Pathway Analysis (IPA; Ingenuity Systems). The list of DEG was uploaded to run the core analysis, focusing on upstream transcription regulators and their downstream target genes. For the metabolomics network reconstruction, the list of significant compounds were annotated against the PubChem biochemical database and used for the IPA analysis.

### KEGG pathway analysis

The dynamic impact approach (DIA) [18] was used for pathway analysis using the Kyoto Encyclopedia of Genes and Genomes (KEGG) database. As with the IPA analysis, the full list of DEG was used. The DIA allows for an assessment of the ‘impact’ (biological relevance) and ‘flux’ (direction of the impact, downregulation or upregulation) within KEGG pathways as a function of the DEG list. The entire DEG also were used in the KegArray tool (http://www.kegg.jp/) to obtain an overall picture of the top 20 metabolic and non-metabolic KEGG pathways that resulted from the DEG. When feasible, the biochemical compounds identified from metabolomics analysis were further annotated with their corresponding pathways.

### Results and discussion

There is a substantial body of information encompassing classical biochemistry and novel omics tools to characterize ketosis [6]. Unlike characterization of the physiologic changes during the disease, few data exist on the profiles of genes, proteins, and/or metabolites in liver tissue that may predispose the cow to developing clinical ketosis. In the present study, transcriptomic and metabolomics were combined to uncover changes occurring at the gene, metabolite, and pathway level. This degree of integration represents an initial attempt to better understand the potential culprits of ketosis development at the liver level before parturition. A clear outcome of the present study was that there were fewer changes at the metabolome than transcriptome level.

A total of 3065 differentially expressed genes in the KET vs. OVE (Additional file 1) were detected at a *P-*value cut-off of ≤0.05 and an FC threshold of ≥ |1.5|. Among these, 2091 were upregulated and 974 were downregulated. By reducing the *P* value cut-off to ≤0.01 and doubling the FC threshold to ≥ |3|, a total of 121 DEG were obtained, of which 22 were upregulated (fold change ≥3) (Table 1) and 39 were downregulated (fold change ≤ − 4) (Table 2). Overall, a mixed response in terms of up and downregulated DEG was observed within metabolism and immune system pathways.

**Table 1:** Differentially expressed genes with a *P*-value < 0.01 and fold change ≥3 in liver tissue harvested 10 days prior to parturition from Holstein cows with (KET) or without (OVE) clinical ketosis during the first week postpartum. Both groups of cows were fed a diet containing 1.54 Mcal/kg of dry matter and 15.0% crude protein from − 21 days prepartum to calving, and a common lactation diet containing 1.75 Mcal/kg of dry matter and 17.5% crude protein

Symbol	Description	KET vs. OVE
*CCL2*	Chemokine (C-C motif) ligand 2 (CCL2)	5.12
*CLCA2*	Chloride channel accessory 2	5.09
*CACNA1D*	Calcium channel, voltage-dependent	4.51
*ADARB2*	Adenosine deaminase, RNA-specific	4.51
*KRT9*	Keratin 9	4.45
*KIFC2*	Kinesin family member C2	4.34
*MARK1*	MAP/microtubule affinity-regulating kinase 1	4.22
*POLE2*	Polymerase (DNA directed), epsilon 2 (p59 subunit)	4.19
*SLC22A2*	Solute carrier family 22 (organic cation transporter), member 2	4.06
*GPR63*	G protein-coupled receptor 63	3.98
*ANO3*	Anoctamin 3	3.88
*XK*	X-linked Kx blood group (McLeod syndrome)	3.83
*DUSP4*	Dual specificity phosphatase 4	3.73
*SPATA7*	Spermatogenesis associated 7	3.64
*ZC3H4*	Zinc finger CCCH-type containing 4	3.59
*SYCE3*	Synaptonemal complex central element protein 3	3.57
*MYRIP*	Myosin VIIA and Rab interacting protein	3.40
LOC787081	Predicted: UPF0632 protein A	3.36
*CNR1*	Cannabinoid receptor 1 (brain)	3.21
*SPATA17*	Predicted: spermatogenesis associated 17	3.20
*PCBP3*	Poly(rC) binding protein 3 (PCBP3)	3.09
*ADAM32*	ADAM metallopeptidase domain 32 (ADAM32)	3.08

**Table 2:** Differentially expressed genes with a *P*-value < 0.01 and fold change ≤ − 4 in liver tissue harvested 10 days prior to parturition from Holstein cows with (KET) or without (OVE) clinical ketosis during the first week postpartum. Both groups of cows were fed a diet containing 1.54 Mcal/kg of dry matter and 15.0% crude protein from − 21 days prepartum to calving, and a common lactation diet containing 1.75 Mcal/kg of dry matter and 17.5% crude protein

Symbol	Description	KET vs. OVE
*DOCK3*	Dedicator of cytokinesis 3	−8.12
*FAM131A*	Family with sequence similarity 131, member A	−6.91
LOC528412	Multidrug resistance-associated protein 4	−6.80
*COBRA1*	Cofactor of BRCA1	−6.24
*CPXM2*	Carboxypeptidase X (M14 family), member 2	−6.13
*UNC13D*	Unc-13 homolog D (*C. elegans*)	−5.18
LOC780781	Keratin associated protein	−5.11
*BTBD10*	BTB (POZ) domain containing 10	−5.09
*BAD*	BCL2-associated agonist of cell death	−5.08
*NEU4*	Sialidase 4	−5.08
*SS18*	Synovial sarcoma translocation, chromosome 18	−4.82
*TAPBPL*	TAP binding protein-like	−4.80
KIAA0922	KIAA0922 ortholog	−4.77
*APOBR*	Apolipoprotein B receptor	−4.68
*PROK2*	Prokineticin 2	−4.64
*WDR6*	WD repeat domain 6	−4.60
*EVC2*	Ellis van creveld syndrome 2	−4.58
*SYN3*	Synapsin III	−4.50
*GLCCI1*	Glucocorticoid induced transcript 1	−4.50
*DLGAP5*	Discs, large (Drosophila) homolog-associated protein 5	−4.47
*CIB2*	Calcium and integrin binding family member 2	−4.47
*TBC1D19*	TBC1 domain family, member 19	−4.41
*ARNT*	Aryl hydrocarbon receptor nuclear translocator	−4.40
*ACPT*	Acid phosphatase, testicular	−4.40
*MAPK3*	Mitogen-activated protein kinase 3	−4.39
*TUBG1*	Tubulin, gamma 1	−4.39
*NCOA2*	Nuclear receptor coactivator 2	−4.29
*REN*	Renin	−4.25
*DST*	Predicted: Dystonin, transcript variant 1	−4.23
*PTK2*	PTK2 protein tyrosine kinase 2	−4.21
*HCN1*	Hyperpolarization activated cyclic nucleotide-gated potassium channel 1	−4.20
*DEFB1*	Defensin, beta 1	−4.16
*TTF2*	Transcription termination factor, RNA polymerase II	−4.13
*NPB*	Neuropeptide B	−4.13
*RAB27A*	RAB27A, member RAS oncogene family	−4.09
*ZYG11A*	Zyg-11 homolog A (*C. elegans*)	−4.08
*MAP3K4*	Mitogen-activated protein kinase kinase kinase 4	−4.06
*TPGS1*	Predicted: Chromosome 7 open reading frame, human C19orf20	−4.04
*C1QTNF3*	C1q and tumor necrosis factor related protein 3	−4.00

**Fig. 1:**
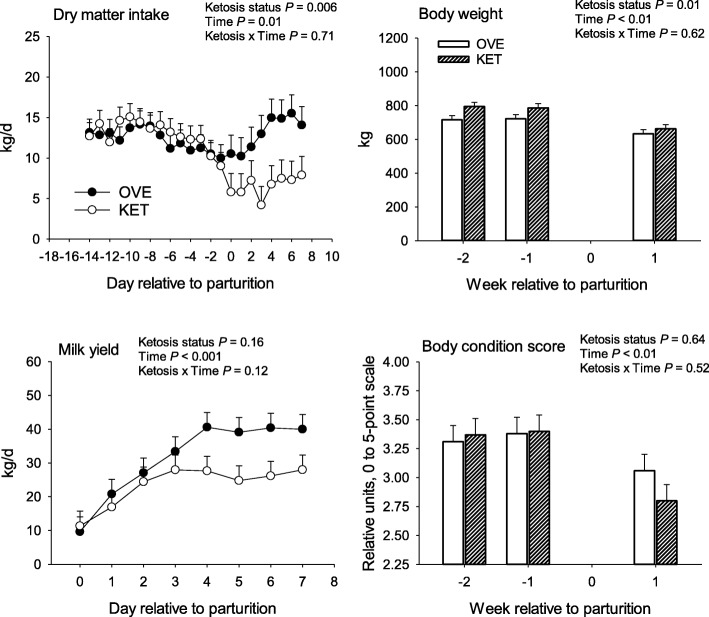
Dry matter intake (DMI), milk yield, body weight, and body condition score in cows overfed energy prepartum that developed ketosis within 7 days postpartum (KET) or remained healthy (OVE). Both groups of cows were fed a diet containing 1.54 Mcal/kg of dry matter and 15.0% crude protein from − 21 days prepartum to calving, and a common lactation diet containing 1.75 Mcal/kg of dry matter and 17.5% crude protein

**Fig. 2:**
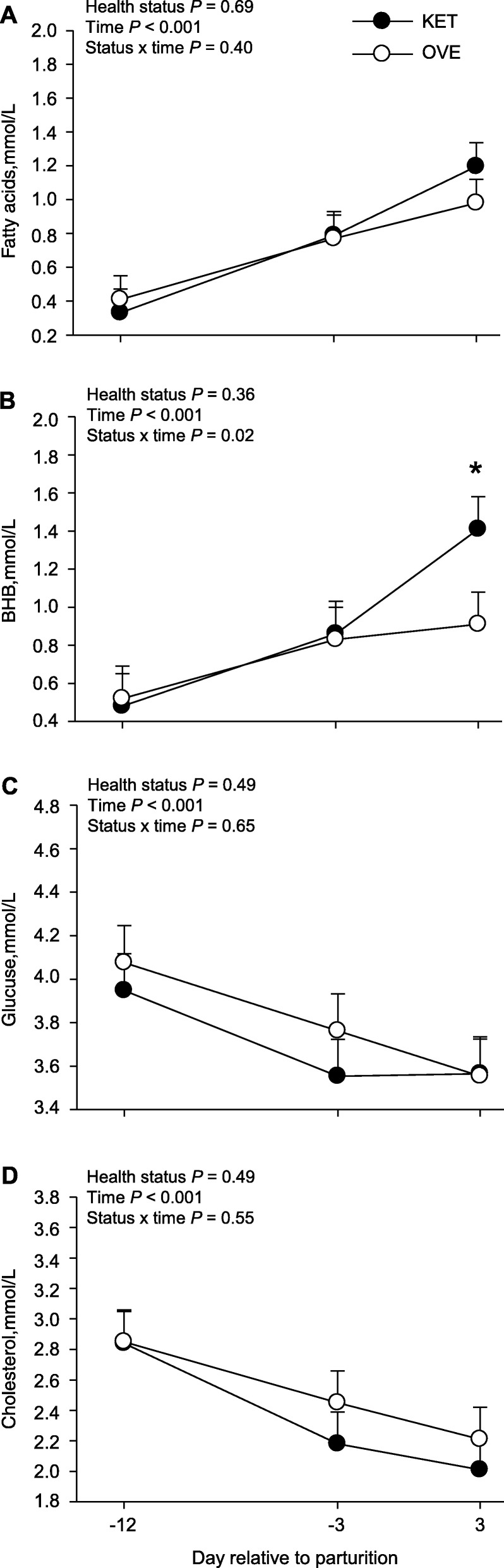
Plasma concentrations of fatty acids (**a**), hydroxybutyrate (BHB; **b**), glucose (**c**), and cholesterol (**d**) around parturition in cows overfed energy prepartum that developed ketosis within 7 days postpartum (KET) or remained healthy (OVE). *Means differ (status × time). Both groups of cows were fed a diet containing 1.54 Mcal/kg of dry matter and 15.0% crude protein from − 21 days prepartum to calving, and a common lactation diet containing 1.75 Mcal/kg of dry matter and 17.5% crude protein

For metabolomics analysis, we used a *P* ≤ 0.10 threshold to determine significantly affected biochemical compounds (Table 4). Using this selection criterion resulted in a total of 18 affected biochemical compounds, of which 4 were upregulated and the remaining downregulated. A full list of 313 biochemical compounds identified can be found in the Additional file 2. The statistical analysis of DMI and milk yield and plasma systemic biomarkers of energy balance, liver function, and inflammation are reported in Fig. 1, Fig. 2, and Additional file 3. Despite KET cows having lower overall (*P* < 0.01) DMI compared with OVE due to the response after parturition, milk production did not differ statistically. The lower DMI postpartum in KET cows was associated with greater (*P* < 0.05) BHB compared with OVE cows, in fact, confirming that KET cows underwent a more severe degree of NEB that eventually led to a clinical condition.

The metabolomics analysis identified biomarkers related to various aspects of lipid, amino acid, and carbohydrate metabolism (Table 4). Among these, cows in KET had greater relative concentrations of lipid-related metabolites such as glycochenodeoxycholate and butyrylglycine along with 1-methylimidazoleacetate from histidine metabolism, whereas various amino acid-related metabolites such as dipeptides, kynurenine, and lipid-related compounds such as 1-palmitoylglycerophosphoglycerol, D-erythro-C16-ceramide (also known as N-palmitoyl-D-erythro-sphingosine), 3-dehydrocarnitine, tetradecanedioate, and 1-oleoylglycerophosphoethanolamine were lower. The phosphatidylcholine intermediate metabolite cytidine 5′-diphosphocholine also was greater in KET cows.

In general, in terms of metabolite categories, the present metabolomics results are somewhat similar to those reported recently in serum of dairy cows that developed clinical mastitis postpartum [19]. It is also noteworthy that the present data are closely related to blood metabolomics comparisons performed to study non-alcoholic fatty liver disease (NAFLD) [20]. For instance, just as in the present study, the concentrations of glycochenodeoxycholate and various other bile acids were markedly greater in humans with NAFLD [20, 21]. Unlike humans with NAFLD, however, cows with KET did not have greater concentrations of carnitine-related metabolites or kynurenine.

### Networks of transcription regulators, biochemical compounds and their integration

**Fig. 3:**
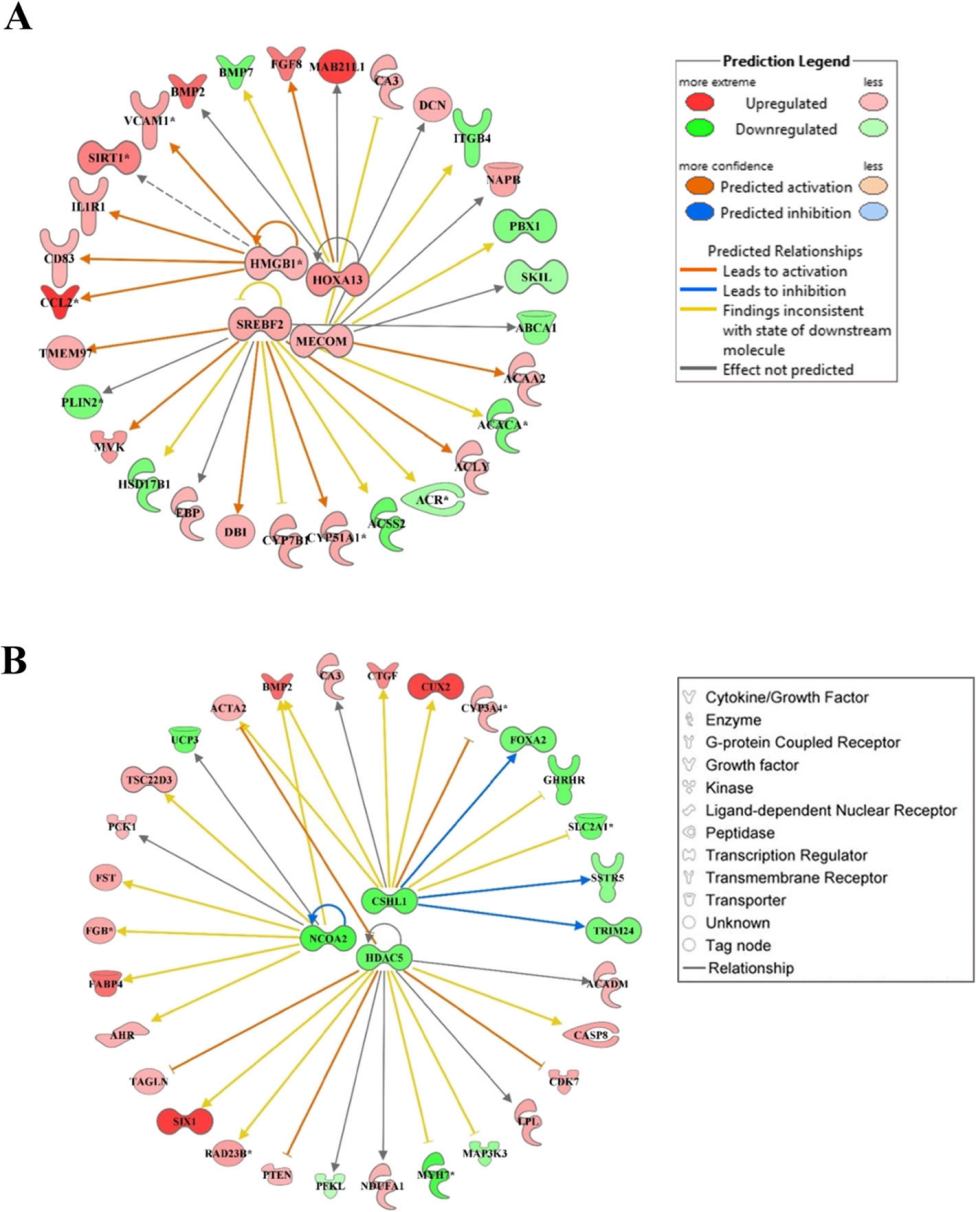
Network analysis using Ingenuity Pathway Analysis (IPA) among 4 upregulated (**a**: *HMGB1*, *HOXA13*, *SREBF2*, *MECOM*) and 3 downregulated (**b**: *NCOA2*, *CSHL1*, *HDAC5*) transcription factors and differentially expressed genes due to onset of ketosis postpartum. Both groups of cows were fed a diet containing 1.54 Mcal/kg of dry matter and 15.0% crude protein from − 21 days prepartum to calving, and a common lactation diet containing 1.75 Mcal/kg of dry matter and 17.5% crude protein

The network analysis for both DEG and biochemical compounds was conducted using IPA software. For DEG, we used the upstream analysis tool in IPA to identify transcription regulators among the DEG (Suppl. File 1). There were in total 9 upregulated and 3 downregulated transcription regulators among the DEG. The network of the 4 upregulated transcription regulators for which IPA identified connections within the list of DEG is shown in Fig. 3a. The upregulated transcription regulators were *HMGB1* (high mobility group box 1), *HOXA13* (homeobox protein Hox-A13), *SREBF2* (sterol regulatory element binding transcription factor 2), *MECOM* (MDS1 and EVI1 complex locus protein EVI1), *NKX2–1* (NK2 homeobox 1), *SATB1* (special AT-rich sequence binding protein 1), *HIF1A* (hypoxia-inducible factor 1-alpha), and *NFE2L2* (nuclear factor erythroid 2-like 2) (Suppl. File 1).

Among upregulated transcription regulators, *HMGB1*, *STAB1* (stabilin 1), and *NFE2L2* are involved in immune response, oxidative stress, and inflammation. Both *HOXA13* and *MECOM* are involved in gene regulation, cell development, differentiation, and proliferation. Hypoxia-inducible factor 1-alpha plays an important role in cellular response to systemic oxygen levels, and glucose metabolism and iron homeostasis. At least in non-ruminants, but also in cows with clinical ketosis [22], *SREBF2* controls cholesterol homeostasis by regulating transcription of sterol-regulated genes. *NKX2–1* also known as ‘thyroid specific enhancer binding protein’ is involved in the regulation of genes associated with the thyroid, lung, and diencephalon.

The downregulated transcription regulators were *CSHL1* (chorionic somatomammotropin hormone-like 1), *NCOA2* (nuclear receptor coactivator 2), *HDAC5* (histone deacetylase 5), *GLI3* (Zinc finger protein GLI3), *SKIL* (SKI-Like proto-oncogene), *SPIB* (Spi-B transcription factor), and *SNAI1* (Snail family zinc finger 1). The network of the top 3 most-downregulated transcription regulators is shown in Fig. 3b. These genes are involved in functions (https://www.ncbi.nlm.nih.gov/gene/) including cell growth control (*CSHL1*), transcriptional activity and cell signaling (*GLI3*, *SNAI1*), transcriptional regulation, cell cycle progression, growth and differentiation (*HDAC5*, *SKIL*, *SPIB*), and nuclear hormone receptors including steroid, thyroid, retinoid, and vitamin D receptors (*NCOA2*).

**Fig. 4:**
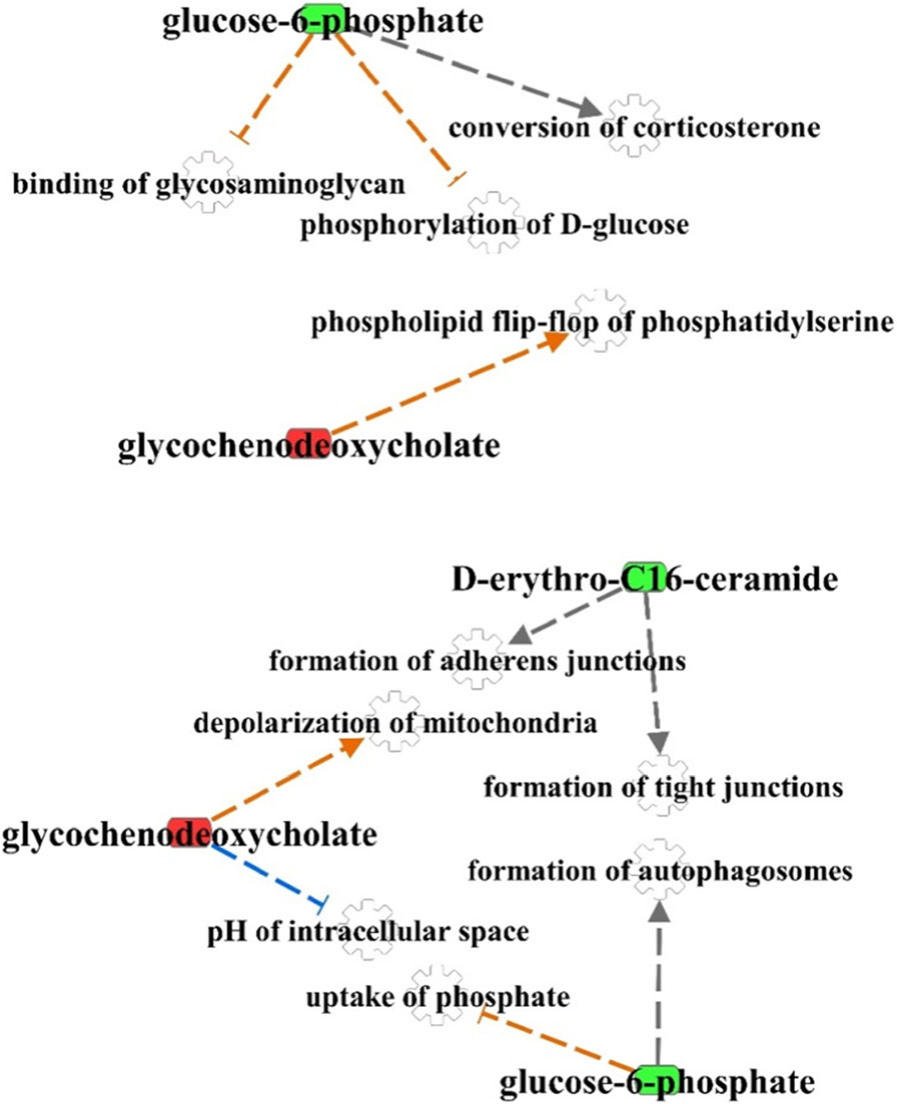
Representation of biochemical compounds in the form of metabolic (A: carbohydrate and lipid metabolism) and non-metabolic (B: cellular functions) networks in cows overfed energy prepartum that developed ketosis within 7 days postpartum (KET) or remained healthy (OVE). Both groups of cows were fed a diet containing 1.54 Mcal/kg of dry matter and 15.0% crude protein from − 21 days prepartum to calving, and a common lactation diet containing 1.75 Mcal/kg of dry matter and 17.5% crude protein

The network analysis between biochemical compounds and biological processes in the IPA knowledgebase is shown in Fig. 4. Among the 13 biochemical compounds significantly affected in KET cows, glucose-6-phosphate, glycochenodeoxycholate, and D-erythro-C16-ceramide could be mapped against known cellular functions in the IPA knowledgebase (Fig. 4).

**Fig. 5:**
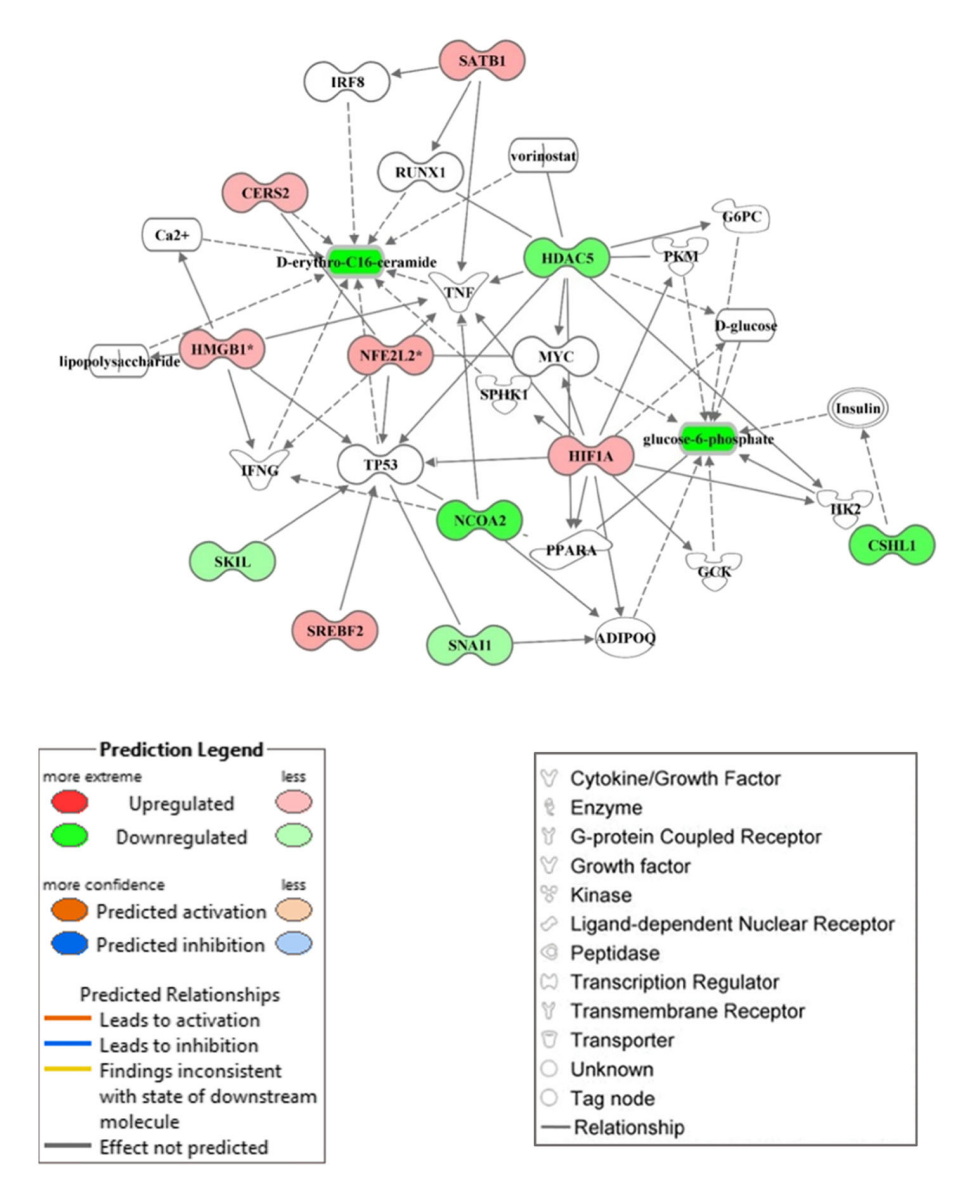
Data integration from transcription regulators and biochemical compounds in cows overfed energy prepartum that developed ketosis within 7 days postpartum (KET) or remained healthy (OVE). Both groups of cows were fed a diet containing 1.54 Mcal/kg of dry matter and 15.0% crude protein from − 21 days prepartum to calving, and a common lactation diet containing 1.75 Mcal/kg of dry matter and 17.5% crude protein

Figure 5 depicts data integration with the IPA knowledgebase using transcription regulators and biochemical compounds identified as significantly affected in cows with KET. The transcription regulators are linked with other potential downstream genes (with white background) and biochemical compounds (with white background) via direct and indirect relationships (according to the IPA legend). Although metabolomics research including nuclear magnetic resonance (NMR), mass spectrometry coupled with either liquid chromatography (LC-MS) or gas chromatography (GC-MS) [23] have been used to different degrees in research with transition cows [24, 25], only few examples of dataset integration exist [26] and none (to our knowledge) focused on the liver.

### KEGG pathways

**Fig. 6:**
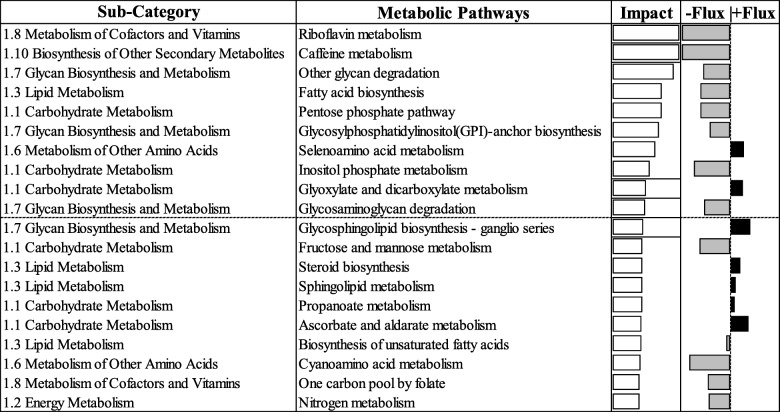
The top 20 most impacted metabolic Kyoto Encyclopedia of Genes and Genomes Pathways ranked by overall impact values in liver of cows overfed energy prepartum that developed ketosis within 7 days postpartum (KET) or remained healthy (OVE). The impact and flux columns are shown on the right hand side. The impact values are represented by transparent bars ranging from 0 to 50. The flux values are represented by grey colored bars ranging from −50 to 0 (− flux) and black colored bars ranging from 0 to + 50 (+ flux) based on the direction of the impact. Both groups of cows were fed a diet containing 1.54 Mcal/kg of dry matter and 15.0% crude protein from − 21 days prepartum to calving, and a common lactation diet containing 1.75 Mcal/kg of dry matter and 17.5% crude protein

**Fig. 7:**
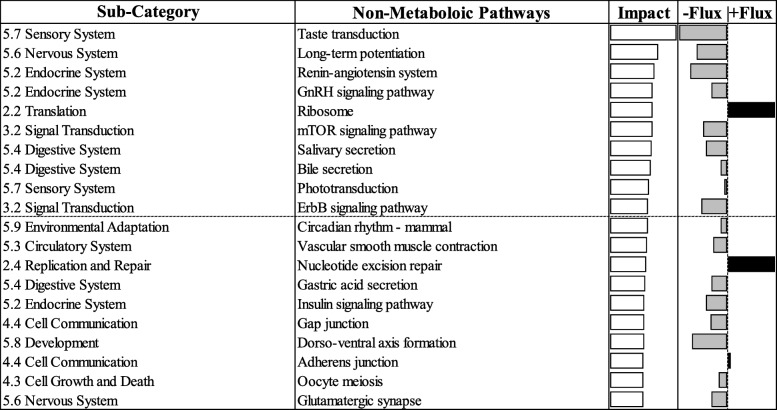
The top 20 most impacted non-metabolic Kyoto Encyclopedia of Genes and Genomes Pathways ranked by overall impact values in cows overfed energy prepartum that developed ketosis within 7 days postpartum (KET) or remained healthy (OVE). The impact and flux columns are shown on the right hand side. The impact values are represented by transparent bars ranging from 0 to 50. The flux values are represented by grey colored bars ranging from − 50 to 0 (− flux) and black colored bars ranging from 0 to + 50 (+ flux) based on the direction of the impact. Both groups of cows were fed a diet containing 1.54 Mcal/kg of dry matter and 15.0% crude protein from − 21 days prepartum to calving, and a common lactation diet containing 1.75 Mcal/kg of dry matter and 17.5% crude protein

Results from the DIA analysis using KEGG with the most-affected biological subcategories are reported in Figs. 6 and 7. Among results from the DIA analysis, the discussion in the sections below concerns ‘metabolic’ (Fig. 6) and ‘non-metabolic’ (Fig. 7) pathways, with a focus on the top 20 most-impacted pathways in cows that developed ketosis. The term “impact” refers to the biological importance of a given pathway as a function of the change in expression of genes composing the pathway (proportion of DEG and their magnitude) [18]. Consequently, the direction of the impact, or flux, characterizes the average change in expression as up-regulation/activation, down-regulation/inhibition, or no change.

## Metabolic pathways

### Carbohydrate metabolism

The downregulation of ‘Pentose phosphate pathway’, ‘Inositol phosphate metabolism’, and ‘Glycolysis / Gluconeogenesis’ with KET (Fig. 6, Additional file 1), and the marked negative effect on various individual genes associated with glycolysis and gluconeogenesis (Table 3) underscores the importance of normal hepatic carbohydrate metabolism to prevent ketosis. The most remarkable effect detected in KET cows was the marked downregulation of fructose-1,6-bisphosphatase 2 (*FBP2*; Table 3) that, despite the modest upregulation of well-known gluconeogenic genes (e.g. *PFKFB2*, *PCCA*, and *PCK1*), would have compromised the ability of the liver to generate glucose during the last stages of pregnancy when cows voluntarily reduce DMI [1]. The lower concentration of glucose-6-phosphate (G6P), xylitol, and ribulose revealed by metabolomics (Table 4) support the inhibition not only of glycolysis/gluconeogenesis, but also pentose metabolism.

**Table 3:** Differentially expressed genes (*P*-value < 0.01 and fold-change ≥ or ≤ 1.5) associated with glycolysis and gluconeogenesis in liver tissue harvested 10 days prior to parturition from Holstein cows with (KET) or without (OVE) clinical ketosis during the first week postpartum. Both groups of cows were fed a diet containing 1.54 Mcal/kg of dry matter and 15.0% crude protein from − 21 days prepartum to calving, and a common lactation diet containing 1.75 Mcal/kg of dry matter and 17.5% crude protein

Symbol	Description	KET vs. OVE	*P* value
*FBP2*	Fructose-1,6-bisphosphatase 2	−8.60	0.003
*PDK4*	Pyruvate dehydrogenase kinase 4	−3.35	0.028
*PDK2*	Pyruvate dehydrogenase kinase, isozyme 2	−2.27	0.018
*PFKL*	Phosphofructokinase, liver	−1.57	0.015
*PFKFB2*	6-phosphofructo-2-kinase/fructose-2,6-biphosphatase 2	1.80	0.047
*PCCA*	Propionyl CoA carboxylase, alpha polypeptide	1.70	0.020
*PDHB*	Pyruvate dehydrogenase (lipoamide) beta (PDHB)	1.51	0.045
*PCK1*	Phosphoenolpyruvate carboxykinase 1 (soluble)	1.51	0.035

**Table 4:** Affected biochemical compounds along with their fold change (FC) values in liver tissue harvested 10 days prior to parturition from Holstein cows with (KET) or without (OVE) clinical ketosis during the first week postpartum. Both groups of cows were fed a diet containing 1.54 Mcal/kg of dry matter and 15.0% crude protein from −21 days prepartum to calving, and a common lactation diet containing 1.75 Mcal/kg of dry matter and 17.5% crude protein.

Compound	Pathway or category	KET vs. OVE	
		FC	*P*-value
Glycylvaline	Dipeptide	−1.41	0.01
Leucylaspartate	Dipeptide	−1.46	0.01
Tyrosylglycine	Dipeptide	−1.30	0.03
Glycylisoleucine	Dipeptide	−1.49	0.03
Xylitol	Pentose metabolism	−1.34	0.04
1-Palmitoylglycerophosphoglycerol	Lysolipid	−1.37	0.06
Glycochenodeoxycholate	Primary bile acid metabolism	2.32	0.06
Glucose-6-phosphate	Glycolysis, gluconeogenesis, and pyruvate metabolism	−1.24	0.07
1-Methylimidazoleacetate	Histidine metabolism	1.62	0.07
D-Erythro-C16-ceramide^a^	Sphingolipid metabolism	−1.24	0.07
1-Nonadecanoylglycerophosphocholine	Lysolipid	−3.79	0.08
3-Dehydrocarnitine	Carnitine metabolism	−1.15	0.08
Ribulose	Pentose metabolism	−1.29	0.09
Butyrylglycine	Fatty acid metabolism (also BCAA metabolism)	1.34	0.09
Tetradecanedioate	Fatty acid, dicarboxylate	−1.40	0.09
Kynurenine	Tryptophan metabolism	−2.12	0.10
1-Oleoylglycerophosphoethanolamine	Lysolipid	−1.54	0.10
Cytidine 5′-diphosphocholine	Phospholipid metabolism	1.17	0.10

^a^Also known as N-palmitoyl-D-erythro-sphingosine

Xylitol is a normal metabolite of the glucuronate-xylulose pathway in mammals, with small amounts being synthesized from glucose in certain tissues (e.g. adipose) [27]. In rodent and human liver, xylitol is metabolized mainly to xylulose 5-phosphate in the pentose phosphate pathway [28]. It is noteworthy that early studies in non-ruminants not only demonstrated that xylitol is gluconeogenic, but also that it is a potent insulin secretagogue [29]. The fact that in the sole published study with dairy cows xylitol infusion increased plasma glucose and decreased BHB without changing plasma fatty acids indicates this compound is biologically relevant in the context of glucose homeostasis [30].

### Lipid metabolism

Among the lipid metabolic pathways within the top 20 most-impacted pathways (Fig. 6), ‘Steroid biosynthesis’ and ‘Sphingolipid metabolism’ were induced in the KET vs. OVE group. The ‘Synthesis and degradation of ketone bodies’ (Additional file 1) was the most induced pathway within lipid metabolism in the ketotic group. Together with the negative alterations in carbohydrate metabolism-related pathways, the results suggest that cows predisposed to developing postpartal ketosis as a result of energy overfeeding prepartum are primed partly through altering hepatic ketone body synthesis.

‘Primary bile acid synthesis’ and ‘Primary bile acid metabolism’ were induced in KET vs. OVE cows (Additional file 1 and Table 3, respectively), a response that agrees with the greater relative concentration of glycochenodeoxycholate (Fig. 4a). Liver is the main site of bile acid production, which in turn helps reduce excessive concentrations of free cholesterol in the liver [31]. The concentrations of bile acids in liver and peripheral blood can be influenced by various factors including synthesis, transport across membranes, secretion, enterohepatic recirculation, liver glycine- or taurine-conjugation, and fecal elimination. Although we did not detect differences in plasma cholesterol between KET and OVE cows (Fig. 2), upregulation of genes involved in cholesterol synthesis/metabolism such as *CYP7B1* and *SREBF2* (Additional file 1) [26] along with the marked downregulation of *APOBR* (Table 2) suggest important alterations not only in the ability of the liver to recycle cholesterol from lipoproteins, but also to use it for synthesis of bile acids.

In the first hepatic transcriptome study of clinical ketosis, we reported a marked downregulation of *SREBF2* [22], which is clearly opposite to the present study, suggesting an effect in the regulation of sterol biosynthesis [32]. At least in non-ruminants, the *SREBF2* transcription regulator encodes the crucial protein controlling target enzymes involved in de novo cellular cholesterol synthesis, with its activation promoting hepatocyte cholesterol accumulation [33, 34]. In humans with NAFLD, hepatic expression of *SREBF2* and its target genes was upregulated, with the degree of SREBF2 activation paralleling the severity of hepatic cholesterol overload [35, 36]. Thus, we speculate that KET cows were prone to accumulate greater levels of cholesterol in the liver in the prepartum, potentially as a result of the downregulation of lipoprotein uptake through *APOBR* [37]. From a mechanistic standpoint, we speculate that the overload of cholesterol in liver prior to parturition might increase the risk of ketosis after parturition because the overall inflammatory status that characterizes this period results in impaired very low density lipoprotein (VLDL) secretion from the liver. A range of thresholds and methods can be used for ketosis diagnosis, for example, detection in milk, blood, and urine samples [38]. Thus, the lack of statistical differences in blood biomarkers related to inflammation and oxidative stress in the present study underscores that other factors such as parity, time at harvest of samples and BCS could influence diagnosis results [39, 40]. Although the various plasma biomarkers of inflammation and oxidant status did not differ (Additional file 3), overall, the present data underscore the potential involvement of lipoprotein and cholesterol metabolism in the liver as important causative factors for ketosis.

### Amino acid metabolism

The greater relative concentration of butyrylglycine and 1-methylimidazoleacetate agrees with the induction of ‘Glycine, serine, and threonine metabolism’ and ‘Histidine metabolism’ in KET cows (Additional file 1). The lower relative concentrations of glycylvaline, leucylaspartate, and glycylisoleucine agree with the induction of ‘Valine, leucine, and isoleucine degradation’. Other induced pathways associated with amino acid metabolism include ‘Cysteine and methionine metabolism’, ‘Arginine and proline metabolism’, ‘Lysine degradation’, ‘Tryptophan metabolism’, and ‘Alanine, aspartate and glutamate metabolism’. It is tempting to speculate, as in metabolomic studies of humans with NAFLD, that activation of amino acid catabolism predisposes cows to ketosis, e.g., by accelerating utilization of essential amino acids close to parturition. This idea is surmised because it is well-established that body protein catabolism increases as parturition approaches, hence, partly explaining the lower concentrations of the various metabolites related to these pathways. Alternatively, an increase in catabolism of amino acids such as cysteine and methionine might be related with the need of these compounds for reactions involving synthesis of antioxidants (e.g. glutathione, taurine, hypotaurine), choline, and the methyl donors S-adenosylmethionine [9, 14, 41]. In addition, those amino acids and intermediates such as choline also participate in lipoprotein metabolism, which would encompass cholesterol and choline [32]. The present data underscore the induction of amino acid catabolism as an important feature of clinical ketosis [8], and it highlights a potential opportunity to enhance supplementation of nutrients in the late-prepartum period that might help alleviate those catabolic events.

### Glycan biosynthesis and metabolism

Glycans are carbohydrate molecules that are either linked with lipids to form glycolipids or proteins to form glycoproteins. These carbohydrate molecules are mainly involved in cellular signaling [42]. Cows in KET had an overall inhibition of ‘Other glycan degradation’, ‘Glycosylphosphatidylinositol (GPI)-anchor biosynthesis’, ‘Glycosaminoglycan degradation’, and ‘O-Glycan biosynthesis’ (Fig. 6), while ‘Glycosphingolipid biosynthesis - ganglio series’ and ‘N-Glycan biosynthesis’ were induced. Because of the alterations in cellular function discussed below, we speculate that inhibition of the glycan-related pathways might have contributed to altered cellular signaling, communication, and extracellular matrix (ECM) [43, 44]. In contrast, glycan-related pathway induction might have been associated with a response to help handle misfolded proteins in the endoplasmic reticulum (ER) [45, 46]. Our previous work with periparturient cows underscored the importance of ER in terms of dealing with stress and inflammation [21].

### Metabolism of cofactors and vitamins

Except for ‘Riboflavin metabolism’ and ‘One carbon pool by folate’ (Fig. 6), the remaining pathways associated with cofactors and vitamins (Additional file 1) were induced in KET cows. Riboflavin is part of flavin mononucleotide (FMN) and flavin adenine dinucleotide (FAD) cofactors, which play a critical role in fatty acid oxidation, TCA cycle, the electron transport chain, production of reduced glutathione, and synthesis of 5-methyltetrahydrofolate [47]. The one-carbon metabolism pathway encompasses the methionine cycle, folate cycle, and transsulfuration pathway, all of which interact to generate purines and thymidylate, remethylate homocysteine to methionine, generate SAM, and synthesize antioxidants [48]. Although the benefit of enhancing the supply of methionine in late-pregnancy in terms of decreasing risk of metabolic disorders has been confirmed [9, 49, 50], it is commonly thought that dairy cows do not require dietary water-soluble vitamins due to the contribution from ruminal microbes. Clearly, these data highlight a number of interrelated pathways whose activity is altered in late-pregnancy in cows that develop ketosis. Further studies should measure activities of some the key enzymes in these pathways to better understand the relationship with nutrition and management of the cow prior to parturition. From the present data, it is unclear if activity and/or flux through the pathways also are compromised in cows that become susceptible to ketosis.

## Non-metabolic pathways

### Translation

‘Ribosome’ was one of only two non-metabolic pathways that was activated among the top 20 most-impacted in KET (Fig. 7). Along with other pathways such as ‘Ribosome biogenesis in eukaryotes’, ‘RNA transport’, and ‘mRNA surveillance pathway’ (Additional file 1), ‘Ribosome’ is involved in cellular protein synthesis and turnover. Several pathways related to folding, sorting, and degradation also were induced, including ‘Protein processing in endoplasmic reticulum’, ‘RNA degradation’, ‘Protein export’, ‘Ubiquitin-mediated proteolysis’, and ‘Proteasome’. Together, these results indicate that genes involved in the various aspects of cellular protein homeostasis within the liver were altered prior to parturition in cows prone to develop ketosis.

The importance of genes associated with protein homeostasis in the context of the normal adaptations of the liver to the onset of lactation have been discussed previously [51, 52]. More recent data have confirmed the biological importance of those networks in the periparturient period. For instance, ER stress induced an unfolded protein response (UPR) that was associated with changes in mRNA expression during the transition period [53, 54]. The differential expression of genes associated with the ER stress and UPR in prepartum cows that are susceptible to ketosis may reflect an inflammatory response. However, the fact that systemic biomarkers of inflammation and oxidant status did not differ between KET and OVE cows suggests the existence of “localized” inflammatory mechanisms. For instance, the expression of chemokine (C-C motif) ligand 2 (*CCL2*) had the highest degree of upregulation in KET cows (Table 1). We have detected alterations in mRNA abundance of *CCL2* in adipose tissue and neutrophils during the periparturient period [55–57]. Furthermore, we were the first to profile immune-responsive genes not only in periparturient liver [51], but also in cows with clinical ketosis [22]. Thus, a linkage between early inflammatory activation within the liver and aspects of cellular protein homeostasis might predispose cows to ketosis, especially when overfed energy prepartum.

### DNA replication

The pathways related to DNA replication such as ‘Nucleotide excision repair’, ‘Base excision repair’, and ‘DNA replication’ were induced in cows with KET (Fig. 7, Additional file 1). The induction of these pathways seems to suggest an increase in liver regeneration and/or cell proliferation. Similar mechanisms of cellular proliferation were altered in cows with ketosis in the early postpartum period [22, 58]. As an example, the transcription regulator *MECOM* (MDS1 and EVI1 complex locus) (Fig. 3a) is involved in regulation of cellular proliferation (*SKIL*) [59] and also transcription of metabolism and bile acid synthesis-related genes (*ACACA*, *ACAA2*) [60]. However, whether such effect would be detrimental to hepatic health and render cows more susceptible to ketosis would require more detailed studies.

### Cellular functions

During metabolic disorders, several cellular functions are affected [22]. In the KET vs. OVE group, the inhibited pathways included the ‘mTOR signaling pathway’, ‘ErbB signaling pathway’, ‘VEGF signaling pathway’, ‘Phosphatidylinositol signaling system’, ‘MAPK signaling pathway’, ‘Calcium signaling pathway’, ‘Hedgehog signaling pathway’, ‘Notch signaling pathway’, ‘Wnt signaling pathway’, and ‘Jak-STAT signaling pathway’. In contrast, the ‘TGF-beta signaling pathway’ was the only induced pathway in cows with KET (Additional file 1).

The inhibition of these various signaling pathways may indicate a lower degree of cell-to-cell communication, and a lack of proper signaling mechanisms that could predispose cows to ketosis. The inhibition of these signaling pathways likely would have led to compromising several important biologic functions including a decrease in cell metabolism (e.g., mTOR, Phosphatidylinositol, Calcium, Notch) [61–63], growth, proliferation and survival (e.g., mTOR, ErbB, MAP Kinase, Hedgehog, Wnt) [64, 65], a reduction in differentiation, apoptosis, and cell motility (e.g., ErbB, MAP Kinase, Phosphatidylinositol, Notch, Wnt) [66, 67], a decrease in vascular development (VEGF) [68] and immune response (Jak-STAT, MAP Kinase) [69, 70]. Unlike the multitude of inhibited pathways, the induction of TGF-beta signaling along with downstream target genes such as *SMAD1*, *TGIF1*, and *BAMBI* (Additional file 1) might have been an indication of enhanced cell proliferation and apoptosis regulation [71], i.e., a counter-regulatory response to maintain normal cellular function.

Ingenuity pathway analysis of functional connections between glycochenodeoxycholate, D-erythro-C16 ceramide and glucose-6-phosphate and cellular function and maintenance categories highlighted important associations (Fig. 4b). For instance, increases or accumulation of glycochenodeoxycholate triggers depolarization of mitochondria leading to hepatocyte apoptosis, and alterations in reactive oxygen species generation [72, 73]. The lower concentration of D-erythro-C16 ceramide (Fig. 4b, and Fig. 5) might suggest a decrease in functioning of adherens and tight junctions, both of which play an important role in epithelial cell to cell adhesion [74]. In that context, it is noteworthy to note that ceramides also are important cell signaling lipid molecules able to participate in the control of apoptosis, growth, differentiation and proliferation [75, 76].

### Immune system

The role of the liver as a responder to the stresses characteristic of the periparturient period is well-known [77]. Hepatic transcriptome analyses have revealed not only metabolic networks, but also immune networks that likely contribute to the immune-responsiveness of the organ [51, 78]. Plasma analyses of inflammation biomarkers such as albumin, ceruloplasmin, serum amyloid A (SAA), haptoglobin and interleukin 6 (IL-6) during the transition into lactation not only help understand the normal course of inflammatory process in dairy cows, but also the potential benefits that nutritional management can have on those processes [79]. The liver synthesizes a number of acute-phase proteins [77], hence, transcriptome analyses of this organ can provide additional information about a localized inflammatory response, especially around calving. More importantly, such data can be used to gauge potential linkages with other important biological adaptations of the liver that occur at the transcriptome level, e.g. alterations in lipid metabolism and mitochondrial function [80, 81].

Among the immune-related pathways, bioinformatics analyses revealed that ‘Antigen processing and presentation’ and ‘Complement and coagulation cascades’ (Additional file 1) were the most induced in KET compared with OVE cows. In addition, among the up-regulated transcription factors, our data suggest that *HIF1A*, *HMGB1*, and *NFE2L2* (Fig. 5) are biologically linked with downstream immune-related genes such as *INFG* and *TNF*. Among these, HIF1A is mainly involved in regulation of cell metabolism, stress, and innate immune response [82]. The protein encoded by *HMGB1* acts as a cytokine to respond against cellular injury, infection, and inflammation [83]. The transcription factor NFE2L2 is an important regulator of oxidative stress and helps control pro-inflammatory responses in non-ruminants [84] and dairy cows [85, 86]. Although the systemic concentrations of immune-related biomarkers did not differ between KET and OVE (Additional file 3), these molecular data suggest that ketosis postpartum might be associated with a localized inflammatory response. As such, if this inflammatory response is not properly controlled it might be a causative factor associated with the various cellular signaling events that were inhibited in cows with KET.

## Conclusions

Despite the lack of difference in dry matter intake prior to parturition, both transcriptome and metabolome analyses of liver tissue revealed altered profiles between KET and OVE cows in late-pregnancy, some of which might contribute to onset of ketosis soon after parturition. The combined data analyses revealed more extensive alterations of the liver transcriptome than metabolome in cows overfed energy during the prepartal period and developing ketosis postpartum. It is noteworthy that, in the present study, clinical ketosis postpartum in response to energy overfeeding prepartum was not only related to carbohydrate and lipid metabolism, but also amino acid and vitamin metabolism. Besides metabolism, impaired cellular immune function and inhibition of important cellular signaling pathways also might play a crucial role in leading to ketosis. The causative link between these tissue-level adaptations and onset of clinical ketosis needs to be studied further.

## Supplementary information

**Additional file 1.** Differentially expressed genes (DEG), overall summary of the bioinformatics analysis using the Dynamic Impact Approach (DIA), and individual categories and subcategories affected by the DEG in cows developing ketosis postpartum.

**Additional file 2.** Biochemical compounds identified by the metabolomics analysis of liver tissue.

**Additional file 3.** Profiles of biomarkers analyzed in plasma, and statistical output.

## Data Availability

The datasets analyzed during the current study are available from the corresponding author on reasonable request.

## Abbreviations

BHB: Hydroxybutyrate
CP: Crude protein
DEG: Differentially expressed genes
DIA: Dynamic impact approach
DM: Dry matter
IACUC: Institutional Animal Care and Use Committee
IPA: Ingenuity pathway analysis
KEGG: Kyoto Encyclopedia of Genes and Genomes
KET: Ketosis
NEB: Negative energy balance
OVE: Control, healthy cows
